# Effect of 12 weeks of complex training on occupational activities, strength, and power in professional firefighters

**DOI:** 10.3389/fphys.2022.962546

**Published:** 2022-08-17

**Authors:** Meng Liu, Kaixiang Zhou, Bin Li, Zhenxiang Guo, Yan Chen, Guozhen Miao, Limingfei Zhou, Haoyang Liu, Dapeng Bao, Junhong Zhou

**Affiliations:** ^1^ Sports Coaching College, Beijing Sport University, Beijing, China; ^2^ College of Sports and Health, Chengdu University of Traditional Chinese Medicine, Chengdu, China; ^3^ Cycling and Fencing Sports Administrative Center Under the General Administration of Sport of China, Beijing, China; ^4^ Department of Physical Education, Nanjing University of Aeronautics and Astronautics, Nanjing, China; ^5^ Hebrew SeniorLife Hinda and Arthur Marcus Institute for Aging Research, Harvard Medical School, Bos-ton, MA, United States; ^6^ Maranatha High School, Pasadena, CA, United States; ^7^ School of Strength and Conditioning Training, Beijing Sport University, Beijing, China; ^8^ China Institute of Sport and Health Science, Beijing Sport University, Beijing, China

**Keywords:** firefighter, physical training, complex training, occupational activities, strength

## Abstract

**Objective:** This study examined the effects of 12-week complex training (CT) programs on professional firefighters’ occupational activities, strength, and power.

**Methods:** Thirty men professional firefighters were randomly assigned to the CT group (*n* = 15) and control group (*n* = 15). The CT group performed complex training and the control group completed resistance training (RT) twice a week over 12 weeks. The occupational activities, strength, and power were assessed at baseline and immediately after the intervention by measuring the performance of 100 m load-bearing run (100 m LR), 60 m shoulder ladder run (60 m SLR), 5 m × 20 m shuttle run (5 m × 20 m SR), 4th-floor climbing rope (4th-floor CR), countermovement jump with arm swing (CMJas), seated medicine-ball throw (SMT), one-repetition maximum bench press (1RM BP), and one-repetition maximum back squat (1RM BS).

**Results:** The results showed that compared to RT, CT induced significantly greater improvements in 60 m SLR (*p* = 0.007), 4th-floor CR (*p* = 0.020), CMJas (*p* = 0.001), and SMT (*p* < 0.001).

**Conclusion:** These findings suggest that CT is a novel intervention with great promise of improving professional firefighters’ occupational activities, strength, and power.

## Introduction

Firefighting is considered to be one of the most dangerous civilian occupations with the involvement of a high physical load (Peterson et al., 2008; Poplin et al., 2016; Cvorovic et al., 2020; Chizewski et al., 2021). The successful completion of firefighting task requires a host of essential functions including cardiovascular function, muscular strength, endurance, power, agility, and flexibility (Dennison et al., 2012; Cornell et al., 2017; Stone et al., 2020; Chizewski et al., 2021). For example, greater muscle strength and power has been linked to better performance of rescue task (Dennison et al., 2012; Lindberg et al., 2013; Pawlak et al., 2015), and lower injury risk (Peterson et al., 2008; Frost et al., 2015; Cornell et al., 2021) in professional firefighters. Therefore, strategies aiming at improving muscular strength and power may help improve the professional performance of these populations.

Resistance training (RT) is the most common method to help improve muscular functions that are important for professional firefighters. Studies have reported that RT can successfully improve physical fitness and occupational, and physical ability in firefighters (Roberts et al., 2002; Peterson et al., 2008). However, the RT is “linear” and requires firefighters to regularly participate in training, leading to low compliance in firefighters who work in an irregular, round-the-clock pattern (Peterson et al., 2008). Thus, a more efficient training protocol with a shorter required length is highly-demanded for firefighters.

Complex training (CT) is a novel strategy that combines resistance training with plyometrics training (RT) or explosive exercises to improve the athlete’s maximum strength and explosive force (Fleck and Kontor, 1986). The CT usually involves the combination of two biomechanically similar exercises with the high-load resistance exercise performed first (e.g., squat at 90% of one-repetition maximum), followed by the low-load plyometrics training (e.g., squat jumps) (Fleck and Kontor, 1986; Thapa et al., 2020). Studies have shown that CT can improve sports performance (Kim and Han, 2016; Abade et al., 2020; Gee et al., 2021). More recently, studies have emerged showing that CT can help the development and maintenance of muscle strength (Wallenta et al., 2016; Jones et al., 2019; Thapa et al., 2020) and power (Jones et al., 2019; Pagaduan and Pojskic, 2020), and sprint ability (Freitas et al., 2017; Abade et al., 2020; Thapa et al., 2020), with shorter and more flexible training schedule and lower risk of injury (Chatzinikolaou et al., 2018). However, the effects of CT on muscular function in firefighters have not been examined.

In this pilot, randomized and controlled study, we aimed to examine the effects of a 12-week training program of CT on the strength, power, and rescue performance of professional firefighters. We hypothesize that, compared to RT, CT would induce significantly greater improvements in strength and power of professional firefighters, as well as their occupational activities.

## Materials and methods

### Subjects

**FIGURE 1:**
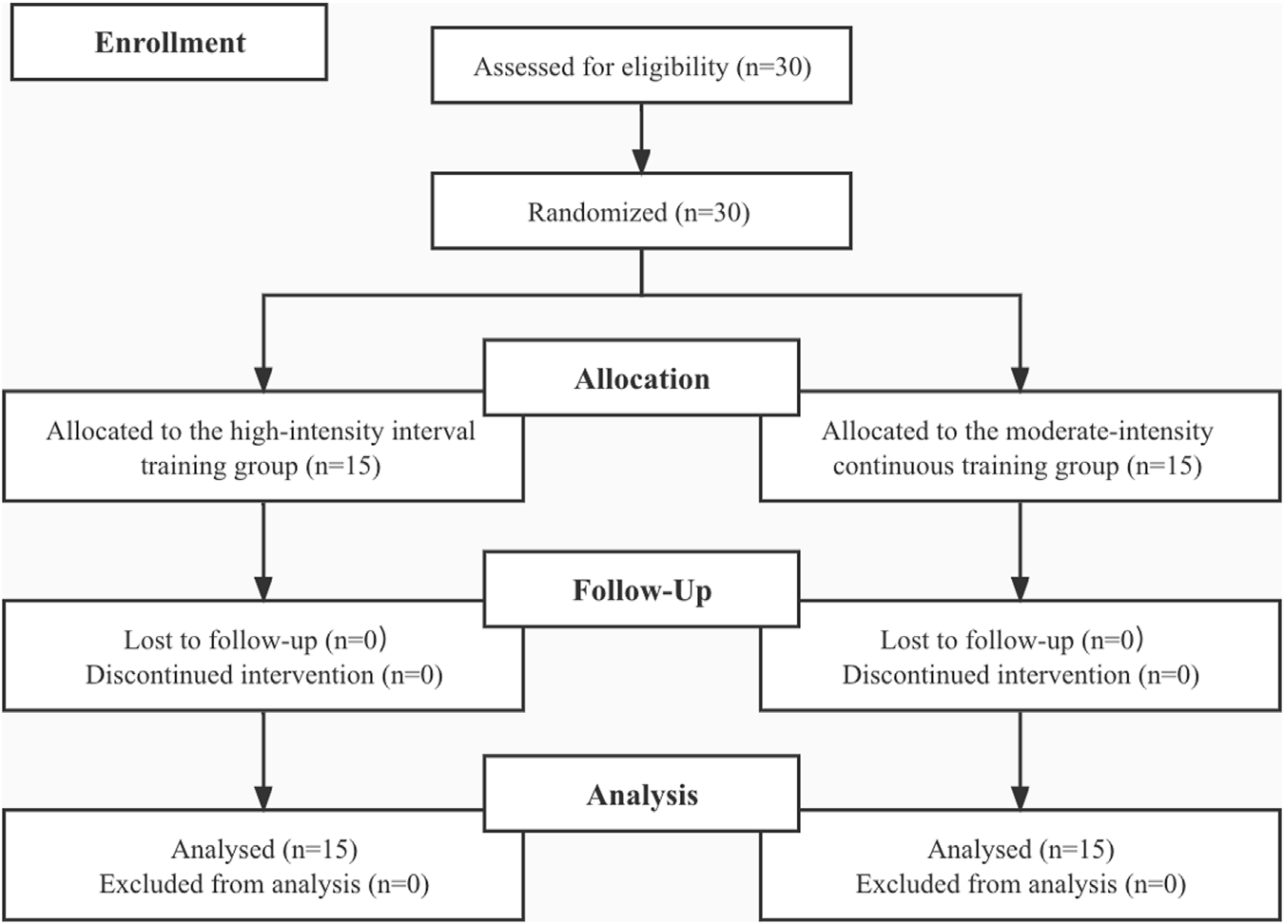
Flow chart of the progress through the phases of the study according to the CONSORT statements.

The sample size of participants (i.e., *n* = 24) was determined using GPower (version 3.1.9.7; Franz Faul, University of Kiel, Kiel, Germany) by using α err prob = 0.05; 1-β Err Prob = 0.8; effect size f = 0.4; test family = *F* test, and statistical test analysis of variance (ANOVA) repeated measures of within-between interaction (Beck, 2013). Therefore, 30 healthy men firefighters were recruited for the study by considering potential drop-out rate of 20%. The participants of firefighters were physically healthy, free from severe injuries, orthopedic or other condition problems that may affect their performance, and with no experience in complex training but with experience in resistance training and plyometrics training. Participants were randomized into CT group (*n* = 15, age: 22.7 ± 1.9 years, height: 176.4 ± 3.1 cm, weight: 69.87 ± 6.4 kg, training experience: 3.9 ± 1.6 years, and work experience: 2.7 ± 1.6 years) and a RT group (*n* = 15, age: 23.9 ± 3.9 years, height: 174.0 ± 4.6 cm, weight: 69.9 ± 6.9 kg, training experience: 4.1 ± 1.3 years, and work experience: 3.2 ± 1.4 years) according to a computer-generated randomization list. The handedness was determined by using Edinburgh Handedness Inventory for their preferred hand in different tasks (e.g., writing, drawing, etc.,) of daily life (Oldfield, 1971). All participants receive the same training except for intervention (See Figure 1 for the firefighter recruitment process). A detailed explanation was provided to the firefighters regarding the aims, benefits, and risks associated with the investigation. Participants were informed that their participation in this study would not affect their employment status. The study protocol was approved by the Beijing Sport University Institutional Research Commission (Approval number: 2022010H), and all procedures were conducted by the Declaration of Helsinki.

### Procedures

All experimental training programs were conducted along with a weekly technical training routine. In the CT group, a 12-week CT intervention consisting of three stages (i.e., 4 weeks per stage) was developed according to the demands of firefighters’ movement patterns and the actual situation of the training site, in addition to the original fire brigade training plan. Eight pairs of training movements, each consisting of one RT and one PT [i.e., total of 16 movements (Supplementary Figure S1)], were implemented (Supplementary Table S1). The training sessions were completed each Monday and Thursday from 7:00 to 9:00 p.m. On each training day, each participant was asked to complete two pairs of CT, as presented in Supplementary Table S1, three times. Considering that the physical status of participants on each training day may differ, we set a range of the number of required repetitions of the movement, which can make the task load similar to each participant. Specifically, within each pair, participants were asked first to perform the RT movement of 4–6 repetitions (reps) and then the PT movement of 10–12 reps. The rest between each movement and between pairs was 4 min. Participants in the RT group completed a 12-week RT intervention. The RT was completed on the same day as CT (i.e., every Monday and Thursday from 7:00 to 9:00 p.m.). On each training day, participants were asked to complete six sets of two RT movements (as presented in Supplementary Table S2), and within each set, participants completed 6–10 reps for each movement. The rest between each set was 4 min.

The one-repetition maximum (1RM) test for each movement was assessed once every 4 weeks to adjust the training plan, following the protocol proposed by Tomchuk (2010). When the training cannot be carried out in special circumstances, such as rescue missions, supplementary training will be completed on Saturday from 8:00 to 10:00 a.m. The two groups completed a standardized 8–15 min warm-up before every training session. The warm-up included low-intensity running, coordination exercises, dynamic movements (lunges and skips), sprints, and dynamic stretching of the lower-limb muscles, and the training load was similar. After the training session, both groups performed a standard cooldown of 8–15 min, consisting of static stretching. All participants were tested 1 week before and within 1 week after the training, and the test sequence, personnel, and location were consistent. The assessment of the capacity for occupational activity was completed using an “in-house” designed physical evaluation index formulated by the Chinese fire department and was selected based upon the situation of the fire scene and the suggestions from firefighter experts. All participants completed all the tests before the baseline and the baseline test 72 h later to assess the test-retest reliability. The experimental process was completed by the fire commander and the external physical trainer. The firefighter’s attendance was recorded, and those who were absent three times or more were excluded from the statistical analysis.

### Test of firefighting occupational activity ability, strength, and power

All strength and power tests were overseen by a National Strength and Conditioning Association (NSCA)-certified strength and conditioning specialist. Each occupational activities tests were performed with maximum speed and maximum effort. The duration of each task time was measured with a hand-held stopwatch (S143, SEIKO, Tokyo, Japan) and recorded to the nearest 100th of a second. Although the participants were familiar with each task, as these tasks are a part of standard pieces of training evolutions within the fire department, we felt it was essential to provide a standardized review of each task. Therefore, each task was explained and demonstrated before initiating the testing process.

#### 100 m load-bearing run

Participants must wear fire uniforms and carry two hose rolls weighing 35 kg to run 100 m at a maximum speed. The test-retest reliability coefficient (intraclass correlation coefficient, ICC) for this test was 0.806.

#### 60 m shoulder ladder run

Participants have to carry a 30 kg ladder on their shoulders to complete 60 m at the fastest speed. The test-retest reliability coefficient (ICC) for 60 m SLR was 0.849.

#### 5 m × 20 m shuttle run

Participants must wear fire uniforms and carry two hose rolls weighing 35 kg to run five 20 m shuttle runs at maximum speed. The test-retest reliability coefficient (ICC) for this test was 0.802.

#### 4th-floor climbing rope

Participants climb ropes about 12 m high with their bare hands, wearing fire uniforms. Test-retest reliability (ICC) for this test was 0.930.

### Countermovement jump with arm swing

The Just Jump System (Just Jump, Probiotics, Huntsville, AL) calculated the height of CMJas. A previous study reported that this Just Jump System had excellent validity and reliability (Intraclass correlation coefficient = 0.96) of the measurement (McMahon et al., 2016). The CMJas test began with the participants standing on the mat with their feet positioned shoulder-width apart. The participants squatted to their self-selected depth before jumping to a maximal CMJas height using a dynamic arm swing (Acero et al., 2012). Participants were advised to wear suitable sports clothing and footwear for the test, and each participant performed three individual jumps, interspersed with a 30-second recovery (Gee et al., 2021). The test-retest reliability coefficient (ICC) for CMJas was 0.946.

### Seated medicine-ball throw

Within a sports hall, the participants sat on the floor with their lower back and shoulder blades touching the wall and with legs outstretched in front, creating a 90° angle from the hips. A 3-kg medicine ball was held in both hands and released with maximal effort, fully extending the arms and elbows from the chest. The participants were required to maintain their initial starting position, with no other movements allowed throughout the throw (Tomchuk, 2010). Each participant performed three individual throws, interspersed with a 30-second recovery. The test-retest reliability coefficient (ICC) for this test was 0.937.

### One-repetition maximum back squat and one-repetition maximum bench press

The back squat exercise was used to determine each individual’s maximal leg strength according to the protocol proposed by Tomchuk (2010), and the bench press exercise was used to determine each individual’s maximal upper limb strength (Tomchuk, 2010). The 1RM represents the maximum weight a participant can lift throughout the full range of motion (90° knee flexion). Before attempting a 1RM trial, participants performed five to six repetitions at a relatively light load (∼40% of their last 1RM test). After that, three to four repetitions were performed at a heavier load (∼70% of their estimated 1RM). Finally, a single repetition was conducted with a load corresponding to 95% of the estimated 1RM. Afterward, participants attempted a single repeat with the perceived 1RM load. If this load was lifted with proper technique, the load was increased by another 1.0–2.5 kg, and the participant attempted another repetition. Failure was defined as a lift falling short of the full range of motion on at least two trials with a 2 min rest between trials. The 1RM was typically determined within four to five trials. The test-retest reliability coefficient (ICC) for this test was 0.957. The 1RM BP test process is the same as the 1RM back squat. The test-retest reliability coefficient (ICC) for this test was 0.946.

The strength and power performance tests were conducted in the afternoon before the test of firefighting occupational activity. The test sequence is CMJas, SMT, 1RM BS, and 1RM BP. On the morning of the second day, the test of firefighting occupational activity ability was conducted by completing 100 m LR, 60 m SLR, 5 m × 20 m SR, and 4th-floor CR. The interval between the tests was between half an hour and an hour. All tests are tested once except for CMJas and SMT, which were tested three times. The highest from three trials of CMJas and SMT was used in the following analysis, respectively.

### Statistical analysis

Experimental data were processed by IBM SPSS statistical software package (version 26.0, Chicago, IL, United States). All data were presented as “mean ± standard deviation" (M±SD). All data were tested for normal distribution using Shapiro-Wilks test. Outliers, defined as studentized residuals greater than three standard deviations from zero, were identified and removed. To examine the effects of the CT on the strength and rescue performance, we firstly performed a two-way repeated-measure ANOVA (group × time). The dependent variable for each model was 100 m LR, 60 m SLR, 5 m × 20 m SR, 4th-floor CR, CMJas, SMT, 1RM BS, and 1RM BP. The model factors were group, time, and their interaction. When a significant interaction was observed, LSD *post-hoc* correction was performed to identify the location of the significance. Secondly, we examined the effects of training (i.e., CT or RT) on the performance within each group by using separate one-way ANOVA models. The model factor was time. Partial *η* ^*2*^ was used to assess the effect size (ES) where the significance was observed, with its strength being interpreted as the following: <0.06 as small, <0.14 as moderate, and ≥0.14 as large (Cohen, 1988). The relative reliability of the test was assessed using the intraclass correlation coefficient of the 1-way random-effects model with single measure ICC. The level of significance was set at *p* < 0.05 for all tests.

## Results

**FIGURE 2:**
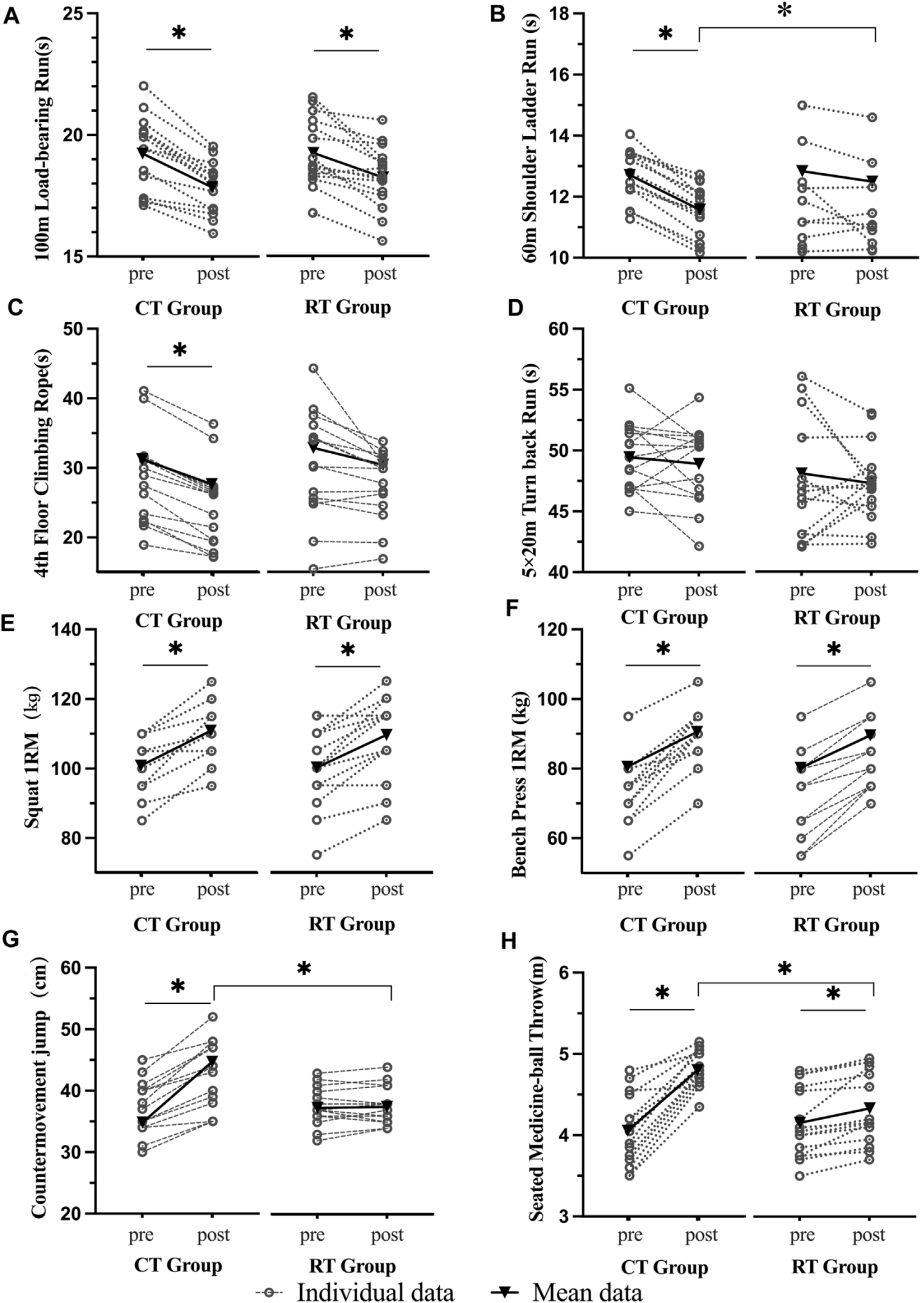
The task performance before and after Complex Training (CT) and Resistance Training (RT). Each spot on the figure reflected one participant, and the mean of each group was presented. Note: *p < 0.05.

All the participants completed this study, and the data obtained from them were used in the analysis. All the data were normally distributed. There was no significant difference in the demographic characteristics, the outcomes of strength and power, and performance between groups (*p* > 0.05). Table 1 presents the descriptive statistics of all tests, results of repeated ANOVA for pre-and post-training fitness testing, and corresponding ESs; Figure 2 shows the individual and mean values for tests before and after CT and RT.

**TABLE 1:** Descriptive statistics of results for CT and RT group before and after the 12-week training intervention.

		CT group				RT group			
		Pre	Post	*p*	Partial $\eta^{2}$	Pre	Post	*p*	Partial $\eta^{2}$
Occupational activity’s ability	100 m LR(s)	19.24 ± 1.53	17.85 ± 1.05*	0.006	0.126	19.25 ± 1.41	18.24 ± 1.30*	0.044	0.071
	60 m SLR(s)	12.71 ± 0.84	11.58 ± 0.84*	0.007	0.123	12.84 ± 1.31	12.50 ± 1.33	0.401	0.013
	5 m × 20 m SR (s)	49.48 ± 2.75	48.92 ± 3.21	0.700	0.003	48.09 ± 5.77	47.30 ± 3.14	0.580	0.005
	4th-floor CR (s)	28.51 ± 6.39	24.41 ± 5.82*	0.020	0.054	30.40 ± 7.69	27.60 ± 4.88	0.226	0.026
Strength and Power	1RM BS (kg)	100.67 ± 7.99	110.67 ± 7.99*	0.007	0.121	100.33 ± 10.93	109.67 ± 11.87*	0.012	0.107
	1RM BP (kg)	73.33 ± 9	90 ± 8.02*	0.000	0.264	74.33 ± 12.52	85.67 ± 10.67*	0.004	0.142
	CMJ (cm)	37.53 ± 4.31	42.53 ± 5.37*	0.001	0.168	37.60 ± 3.09	37.80 ± 3.03	0.893	0.000
	SMT (m)	4.06 ± 0.43	4.80 ± 0.22*	0.000	0.327	4.16 ± 0.43	4.33 ± 0.45*	0.223	0.026

*Statistically significant difference between pre-and post-test, *p* < 0.05.

### Occupational activities performance

The primary two-way repeated-measures ANOVA models revealed no significant interaction of group and time on 100 m LR (*p* = 0.582), 60 m SLR (*p* = 0.173), 5 m × 20 m SR (*p* = 0.905), and 4th-floor CR (*p* = 0.548). Only significant effects of time on 100 m LR (*p* = 0.001), 60 m SLR (*p* = 0.013), and 4th-floor CR (*p* = 0.020).

Secondarily, one-way ANOVA models showed that 100 m LR compared to pre-intervention were significantly decreased after the intervention within both CT (*p* = 0.006) and PT (*p* = 0.044) groups. This showed that both CT and RT could improve the 100 m LR performance. However, the CT (Partial $\eta^{2}$ = 0.126) had a larger ES than the RT (Partial $\eta^{2}$ = 0.071), indicating that CT training may induce larger effects. Meanwhile, 60 m SLR and 4th-floor CR were significantly improved after intervention (60 m SLR: *p* = 0.007; 4th-floor CR: *p* = 0.020) within the CT group, while RT did not induce such significant improvement.

### Strength and power

The primary two-way repeated-measures ANOVA models showed significant interactions between group and time on CMJas (*p* = 0.026) and SMT (*p* = 0.007) but not on 1RM BS (*p* = 0.896) and 1RM BP (*p* = 0.315). The *post-hoc* analysis revealed that the CMJas [F (1,56) = 11.346, *p* = 0.016, *Partial η^* ^*2*^ = 0.099] and SMT [F (1,56) = 7.917, *p* = 0.000, *Partial η^* ^*2*^ = 0.271] in CT group were significantly greater after the CT intervention as compared to all the other pre- and post-interventions. This suggests that CT can better improve the participant’s CMJas and SMT performance than RT.

For 1RM BS and 1RM BP, the secondary one-way ANOVA models showed there were significant improvement in both the CT [1RM BS: *p* = 0.007; 1RM BP: *p* = 0.000] and RT [1RM BS: *p* = 0.012; 1RM BP: *p* = 0.004]. This showed that both CT and RT could improve the 100 m LR performance.

## Discussion

To the best of our knowledge, this is the first study to examine the effects of CT and RT on occupational activities, strength, and power in professional firefighters. This study demonstrated that CT can significantly enhance greater power (CMJas and SMT) performance and thus further improve occupational activities in professional firefighters compared to RT. These suggest that CT is a promising strategy to improve the functionality of professional firefighters and potentially help their occupational activities.

It is observed that compared to the intervention using RT, 12-week CT can significantly improve the power of professional firefighters as assessed by using the task of CMJas and SMT, and there was no significant difference in absolute maximal strength. For firefighters, absolute maximal strength is essential because it provides the force production necessary to lift, hoist, and carry/drag heavy loads (Peterson et al., 2008). Moreover, greater muscular strength is necessary for optimal joint health and integrity and to decrease the risk of accident-related injuries (Peterson et al., 2008; Freitas et al., 2017). This study showed that both CT and RT could significantly increase 1RM BP and 1RM BS without a significant difference between CT and RT, revealing that both CT and RT can improve the maximum strength of firefighters. However, muscle power is also a critical factor in ensuring excellent function for all occupations or endeavors that rely on physical training in emergencies. The increase in nerve impulse and energy production allows firefighters to perform the same task in a shorter time or a greater magnitude of work simultaneously. Due to the power being related both to force generation and movement velocity, the ability to apply muscle power should be regarded as a critical research method and training goal of a specific firefighter training program. Increasing strength is the key to improving performance and speed in training. It is also the key to reducing physical energy consumption in mid-to-high endurance (Peterson et al., 2008; Freitas et al., 2017). The results in this study showed a significantly more significant increase in CMJas and SMT after CT training compared to RT at the end of the 12-week training program, revealing that CT would be more appropriate for improving firefighter’s power compared to RT. Our results are consistent with previous studies showing the longer-term effects of CT on athletes’ lower-body and upper-body power (Matthews et al., 2010). In RT, the muscle isotonic contraction speed is slow, and the conversion time between eccentric contraction and centripetal contraction is longer (Padulo et al., 2012). Weakley et al. (2021) analyzed the speed at the completion of a single set of bench press exercises for subjects of different levels (including weightlifters) in multiple maximum strength training experiments. They found that the speed of the bench press was about 0.17 m/sec. Obviously, the strength exercise method with slower muscle contraction cannot meet the firefighter’s demand for explosive power when completing tasks. Therefore, on the RT exercises, enhanced exercises are added to form a complex training program, which not only makes up for the shortcomings of traditional RT with insufficient muscle contraction speed (Thapa et al., 2020) but also reinforcement exercises are added to different strength training stages to adjust the changes in strength training load intensity (Pagaduan and Pojskic, 2020). According to the strength-speed curve, a heavier load (above 75% 1RM) in strength training will enhance the high-strength part of the curve, while a low-load (50% 1RM or less) fast-moving exercise will affect the strength-speed curve high-speed area (Haff and Nimphius, 2012). The CT fully uses the characteristics of heavy-load slow-strength training and low-load fast-reinforcement activities so that the entire strength-speed curve has a more comprehensive adaptive change, and the effect is better than pure heavy-weight RT.

The increase in firefighters’ maximum strength and power has improved the rescue ability of firefighters’ occupational activities. The results in this study showed that compared to RT, CT induced a significantly more significant increase in 60 m SLR and 4th-floor CR after CT as compared to RT. Additionally, post-training, there was a significant within-group increase in 100 m Load-bearing Run for the CT and RT group. Still, CT has considerably more excellent ES, revealing that CT would be more appropriate for improving occupational activities than traditional RT. The potential physiological mechanism of compound training that is more in line with the suitability requirements of firefighters is that it utilizes post-activation potentiation (PAP) (Hodgson et al., 2005; Santos and Janeira, 2008; Bauer et al., 2019; Pagaduan and Pojskic, 2020; Garbisu-Hualde and Santos-Concejero, 2021), which increases the phosphorylation of the myosin light chain after the firefighter resists the stimulus with a large load. To make the muscle filaments more sensitive to calcium, reduce presynaptic inhibition, increase the number of cross-bridges, and increase muscle contraction. At the same time, the process of force transmission to the tendon will make the angle between the tendon and the muscle fiber “feather angle” smaller. To improve mechanical transmission efficiency, the whole process activates more motor units, recruits more fast-twitch fibers, and improves the power output of subsequent reinforcement exercises (Hodgson et al., 2005). On the other hand, CT can make full use of the lengthening-shortening cycle (SSC) in reinforced exercises to improve the ability of firefighters to quickly transform from the eccentric muscle contraction phase to the heart contraction phase. This ability is consistent with the muscle contraction mechanism of rescue missions (Peterson et al., 2008).

The cycle of CT and RT in this study gradually increases the intensity of exercises according to the purpose of training at different stages. The increase of the enhanced exercise load refers to the safe load range recommended by the American Physical Fitness Association. In addition, the training program of CT in this study follows the recommendations that the compound training frequency is 1–3 times a week, and the 48–72 h of recovery time should be arranged for the needs of the same muscle group (Ebben and Watts, 1998; Arazi et al., 2014). The training program and training movements of RT in this study are the same as those of the RT program and movements used in the daily training of firefighters. In summary, implementing a CT program in the practice of short-term periodic physical training can adjust the strength of the firefighters’ strength training load intensity, fully optimize the strength-speed curve to increase the maximum strength and power, and shorten the muscle elongation-shorten the cycle. Eventually, a significant increase in the rescue performance of firefighters will be realized.

There are some limitations to this study. First, the study did not include women, and the age range is relatively narrow, focusing on only early-career firefighters. Future studies consisting of a larger number of participants with a balanced sex and border age range are needed to examine and confirm the observations in this study. Second, this study implemented a 12-week intervention with only one follow-up assessment. The optimal intervention intensity and the dose-response relationship between the intervention’s performance and “dose” are still unknown. In addition, although the selection of CT group movements has considered the occupational characteristics of firefighters, movements that are more similar to the occupational activities of firefighters may further improve training effects. Future studies with multiple visits of assessment and with longer-term follow-up periods are needed to determine the “dose-response” relationship between CT on performance. Future studies also need to compare different CT training programs consisting of varying training movements, which will ultimately help the optimized design of the CT intervention. Further limitations also include the order of tests. While the order of tests was conducted based on best practice to reduce the impact of fatigue, performing all tests in a single session may hurt performance. Furthermore, we cannot rule out that the new training method (CT) may lead to stronger training motivation than the traditional training (RT), resulting in a better training effect. Nevertheless, this pilot study showed that CT might strengthen strength and power and improve professional firefighters’ occupational activities. The knowledge obtained from this study will ultimately help inform the design of future larger-scale studies to confirm the findings here.

## Conclusion

This pilot study showed that CT is of great promise to induce significantly greater improvements in strength and power of firefighters compared to RT, thereby better enhancing their capabilities for occupational activity. The knowledge obtained from this study will ultimately help inform the design of future larger-scale studies to confirm the findings in this study and help firefighter agencies to develop more appropriate fitness training and management programs for firefighters in their daily routine.

## Data Availability

The original contributions presented in the study are included in the article/Supplementary Material, further inquiries can be directed to the corresponding authors.
