# Supplementary material for: Effect of 12 weeks of complex training on occupational activities, strength, and power in professional firefighters
Source: Front Physiol. 2022 Aug 17;13:962546. doi: 10.3389/fphys.2022.962546 (PMC9428679; doi:10.3389/fphys.2022.962546)
Supplement: Supplementary file 1 [file DataSheet1.docx]

Supplementary Material

**Table S1. Complex Training Program Protocol Performed by the** **Complex-Paired Training Group**

|  | | **Complex pair** | **Intensity** | | | **Sets*** **repetitions** | **Rest（min）** |
| --- | --- | --- | --- | --- | --- | --- | --- |
|  |  |  | **The ﬁrst stage (1–4 weeks)** | **The second stage (5–8 weeks)** | **The third stage (9–12 weeks)** |  |  |
| The first and second weeks | Monday | Squat + Squat Jump | 75%1RM+ME | 80%1RM+ME | 85%1RM+ME | 3*（4～6+10～12） | 4min |
|  |  | Barbell bench press + High-five push-ups | 75%1RM+ME | 80%1RM+ME | 85%1RM+ME | 3*（4～6+10～12） | 4min |
|  | Thursday | Deadlift + High pull | 75%1RM+50%1RM | 80%1RM+50%1RM | 85%1RM+50%1RM | 3*（4～6+10～12） | 4min |
|  |  | Loaded pull-ups +Elastic band pull-down | 75%1RM+ME | 80%1RM+ME | 85%1RM+ME | 3*（4～6+10～12） | 4min |
| The third and fourth weeks | Monday | Weight-bearing lunge + Split-leg squat jump | 75%1RM+ME | 80%1RM+ME | 85%1RM+ME | 3*（4～6+10～12） | 4min |
|  |  | Dumbbell bench press + Kneeling forward medicine ball | 75%1RM+ME | 80%1RM+ME | 85%1RM+ME | 3*（4～6+10～12） | 4min |
|  | Thursday | Military press + Push press | 75%1RM+50%1RM | 80%1RM+50%1RM | 85%1RM+50%1RM | 3*（4～6+10～12） | 4min |
|  |  | Reverse grip loaded pull-ups + Elastic band pull-up | 75%1RM+ME | 80%1RM+ME | 85%1RM+ME | 3*（4～6+10～12） | 4min |

Note:1RM, 1-repetition maximum; ME, maximal effort; Loaded pull-ups, use the belt to hang the barbell plate to increase the load; Elastic band pull-down and Elastic band pull-up, the first stage uses about 35 pounds of the elastic band, and the second and third stages add 5 pounds to the previous stage; Weight-bearing lunge, use dumbbells to increase the load, alternating feet; Kneeling forward medicine ball, facing the wall, throw the medicine ball forward with both hands, the first stage uses an 8kg medicine ball, and the second and third stages add 2kg to the previous stage.


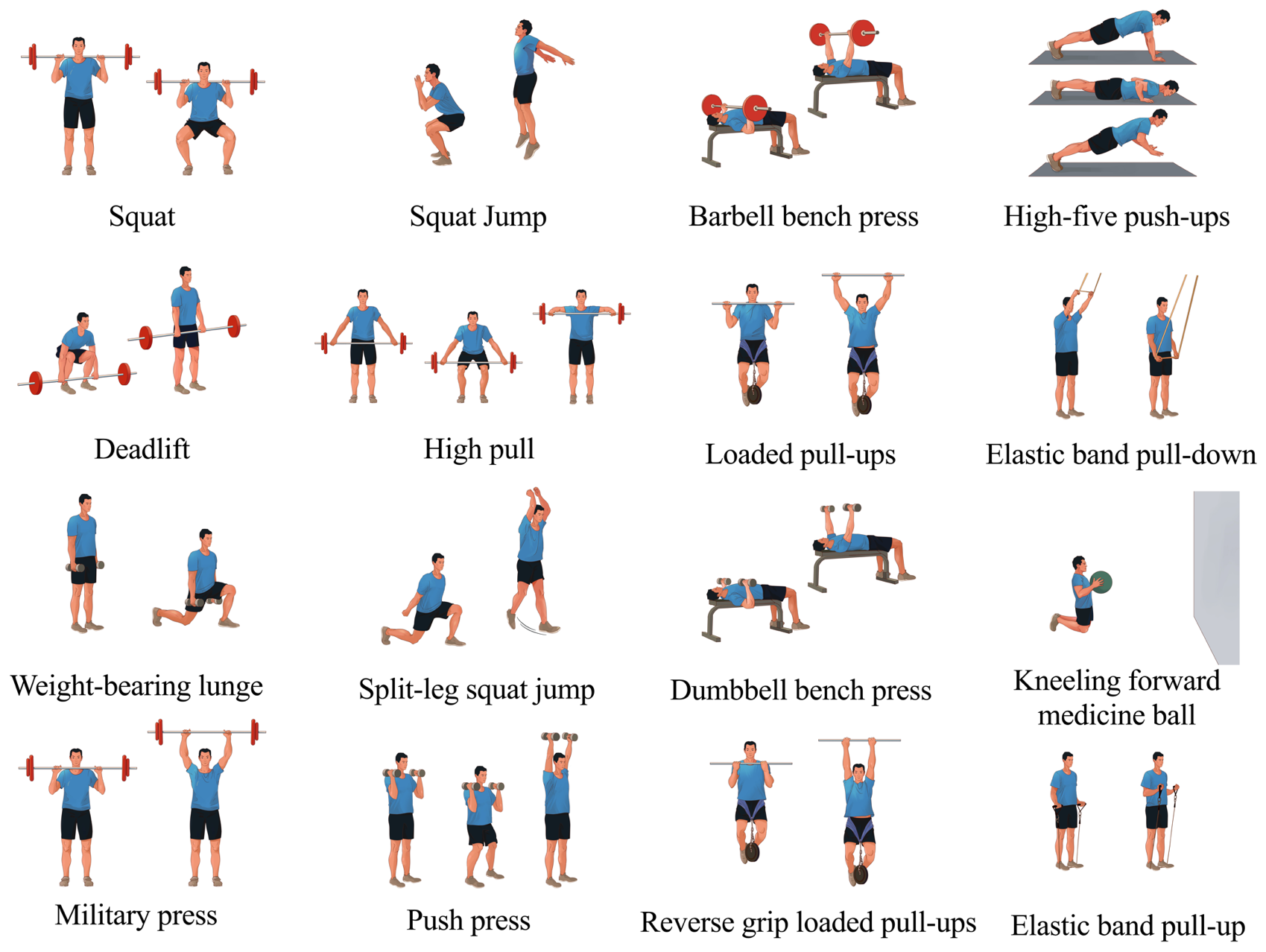


**Supplementary Figure 1.** CT group training movements.

**Table S2.** **Resistance-Training Program Protocol Performed by the Resistance Training Group**

|  | | **Resistance-Training** | **Intensity** | | | **Sets* repetitions** | **Rest（min）** |
| --- | --- | --- | --- | --- | --- | --- | --- |
|  |  |  | **The ﬁrst stage (1–4 weeks)** | **The second stage (5–8 weeks)** | **The third stage (9–12 weeks)** |  |  |
| The first and second weeks | Monday | Squat | 75%1RM | 80%1RM | 85%1RM | 6*（6～10） | 4min |
|  |  | Barbell bench press | 75%1RM | 80%1RM | 85%1RM | 6*（6～10） | 4min |
|  | Thursday | Deadlift | 75%1RM | 80%1RM | 85%1RM | 6*（6～10） | 4min |
|  |  | Loaded pull-ups | 75%1RM | 80%1RM | 85%1RM | 6*（6～10） | 4min |
| The third and fourth weeks | Monday | Weight-bearing lunge | 75%1RM | 80%1RM | 85%1RM | 6*（6～10） | 4min |
|  |  | Dumbbell Bench Press | 75%1RM | 80%1RM | 85%1RM | 6*（6～10） | 4min |
|  | Thursday | Military press | 75%1RM | 80%1RM | 85%1RM | 6*（6～10） | 4min |
|  |  | Loaded pull-ups | 75%1RM | 80%1RM | 85%1RM | 6*（6～10） | 4min |

Note: 1RM, 1-repetition maximum; ME: maximal effort
